# Nutrient Patterns and Risk of Polycystic Ovary Syndrome

**Published:** 2019

**Authors:** Ghazaleh Eslamian, Azita Hekmatdoost

**Affiliations:** 1- Student Research Committee, Shahid Beheshti University of Medical Sciences, Tehran, Iran; 2- Department of Clinical Nutrition and Dietetics, Faculty of Nutrition and Food Technology, Shahid Beheshti University of Medical Sciences, Tehran, Iran; 3- National Nutrition and Food Technology Research Institute, Shahid Beheshti University of Medical Sciences, Tehran, Iran

**Keywords:** Macronutrients, Micronutrients, Nutrient patterns, Polycystic ovary syndrome, Principal component analysis

## Abstract

**Background:**

There are limited data on the role of nutrient patterns in development of polycystic ovary syndrome (PCOS). The aim of the study is to document the relationship between nutrient patterns and PCOS.

**Methods:**

In this study, 281 incident PCOS women and 472 controls were interviewed through the endocrine clinics between February 2013 and March 2015 in Tehran, Iran. Usual dietary intakes were obtained using a validated semi-quantitative food frequency questionnaire. Factor analysis was conducted on the basis of 32 nutrients. Unconditional logistic regression was performed to ascertain odds ratios. The p<0.05 was considered for significance level.

**Results:**

In principal component analysis two nutrient patterns emerged. Factor 1 had high loadings for riboflavin, niacin, pyridoxine, thiamin, magnesium, pantothenic acid, cobalamin, vitamin C, folate, vitamin D, total fiber, selenium, phosphorus, vitamin E, manganese, vitamin K, monounsaturated fatty acids, polyunsaturated fatty acids, potassium and vegetable protein. Factor 2 characterized by high loadings for carbohydrate, animal protein, fat, cholesterol, saturated fatty acid, sodium, biotin, copper, iron, fluoride, zinc, and calcium. After adjusting for potential confounders, the risk of PCOS was significantly higher in the highest tertile of factor 2 (OR: 2.38, 95% CI: 1.69–3.21). Conversely, being in the highest tertile of factor 1 was associated with a lower risk of PCOS (OR: 0.48, 95% CI: 0.21–0.82).

**Conclusion:**

Our results provide a possible new insight into the interactions between nutrient intakes and PCOS.

## Introduction

Polycystic ovary syndrome (PCOS) is one of the most commonly encountered endocrinopathy in women of reproductive age, with metabolic and reproductive consequences, including anovulation, infertility and increased risk of type 2 diabetes mellitus, hypertension, impaired glucose tolerance (IGT), coronary artery disease, endometrial and perhaps breast cancer (1–3), with an incidence rate of 6–10% (4).

Existing epidemiological studies have illustrated a link between PCOS and lifestyle patterns (5–7). A possible association between diet and PCOS risk is commonly reported (8), whereas the association between nutrient intakes and development of PCOS has not been thoroughly explored (9). Current evidence recommend a diet low in saturated fat and high in fiber from predominantly low glycemic index carbohydrate foods as an optimal diet for PCOS patients (10–12).

Although nutrition has consistently been found to be an important determinant of the PCOS risk, only few studies have evaluated the effect of isolated nutrients on PCOS characteristics (1, 12–15), and our current understanding of the impact of overall nutrient intakes in the etiology of this syndrome is still limited. Nutrient pattern assessment provides us a snapshot of the interactions among different dietary nutrients (16), this might be different from the effects of each one intake separately. Therefore, this study was designed to determine if there is a relationship between the dietary nutrient patterns and PCOS risk.

## Methods

### Participants:

In this case control study, the consecutive incident PCOS cases (n=288) and healthy controls (n=491), aged 20–35 years, were recruited between February 2012 and March 2014 from the endocrinology clinics of hospitals, Tehran, Iran.

Newly diagnosed PCOS cases (identified within 3 month of diagnose) were diagnosed by at least two of the three symptoms according to the international Rotterdam Criteria 2003 (17). Criteria to make the diagnosis of PCOS were included oligo-ovulation or anovulation, clinical or biochemical signs of hyper-androgenism, and polycystic ovaries by Ultrasonography. Controls were randomly selected from hospital patients during the same time period. They were admitted to the same hospitals as the cases’ for a wide spectrum of diseases such as ear/nose/throat diseases, elective surgeries or orthopedic problems, did not have any history of special diet and had regular menstrual cycles (26–33 day cycles). None of the participants were on any special nutritional diet. Neither group suffered from any nutrition-related conditions such as cardiovascular diseases, diabetes, osteoporosis, renal disease or cancers and was on any medication which might affect hormone metabolism. All participants signed the informed consent form. The design of this study was approved by the ethics committee of Shahid Beheshti University of Medical Sciences.

### Socio-demographic, anthropometrics and physical activity:

Using pre-structured pre-tested questionnaires, informed and consenting patients in case and control groups were interviewed face-to-face by trained professional interviewers. Socio-demographic information on age, age at menarche, familial history of PCOS, smoking status, education, history of medication and supplement use and monthly family income was obtained from responses to the pretested baseline questionnaire. Consumption of alcohol and opium was not responded in the present study due to subjects’ cultural and religious believes.

Weight was measured while participants were minimally clothed and standing on digital scales (Soehnle, Berlin, Germany) without shoes and was recorded to the nearest 100 grams. Height was measured in a standing position without shoes while the shoulders were in a normal state using a non-stretch tape meter fixed to a wall and was recorded to the nearest 0.5 *cm*. Weight was then divided by the square root of height for calculation of body mass index (BMI). Waist circumference (WC) was measured at the umbilical site using an outstretched tape meter and without pressure to body surfaces, and was recorded to the nearest 0.1 *cm*. All measurements were carried out by one examiner to avoid random observer error.

Physical activity was measured through interviews using a previously validated questionnaire (18, 19). This questionnaire is consisted of nine different metabolic equivalent (MET) categories on a scale ranging from sleep/rest (0.9) to high-intensity activities (>6). Multiplying the time spent in each activity by the MET value corresponding to that activity, MET *hr* for an activity was calculated. Crude MET *hr/day* was then calculated adding the MET *hr* values for different activities in a day.

### Dietary assessment:

Trained dietitians collected dietary data using a validated semi-quantitative food frequency questionnaire (FFQ) which assessed dietary intakes of cases one year before diagnosis and controls one year before. The validated FFQ consisted of 168 food items, including some of the most common Iranian meals (20). The details of the dietary assessment have been reported previously (15).

Totally, seven cases and eleven controls were excluded from the analysis since their log scales of total energy intake was either >3 or <3 SD from the mean, indicating under/over-reporting. After excluding the under- and over reporters of energy, 281 PCOS cases and 472 controls were included in the analyses. Participation rates were 97.5% and 96.3% among cases and controls, respectively.

### Statistical analysis:

In the present study, “principal components method for factor analysis” of the SPSS (IBM, Armonk, NY) statistical analysis soft-ware version 20 was employed to derive potential nutrient patterns on the basis of 32 nutrients (21). The correlation matrix among the 32 nutrients was visually and statistically examined to justify undertaking factor analysis. Sampling adequacy and intercorrelation of variables were confirmed by Kaiser-Meyer-Olkin score=0.76 and Chi-square for Bartlett’s test of sphericity <0.001, respectively.

Using the correlation matrix, the orthogonal varimax rotation method was applied to achieve a simpler structure facilitating interpretation. The number of meaningful components to retain from the total number of extracted patterns depended mainly on the assessment of scree plots and components’ interpretability. Factor score of each nutrient pattern was considered for each patient by summing the consumption of nutrients with weights that were proportional to their factor loadings. Factor loadings are correlation coefficients between nutrient and nutrient patterns; a positive loading in a factor shows a direct correlation with the factor, whereas a negative loading shows that the nutrient is inversely correlated with the factor. Scores for two nutrient patterns identified in this study (factor 1 and 2) were then divided into three categories based on the tertile.

Chi-square test was employed to evaluate the differences in distribution of categorical variables, and independent t-test was performed to check the differences in distribution of continuous variables. Unconditional logistic regression was performed to ascertain odds ratios (ORs) with 95% confidence interval (CI). The minimal adjusted model included age (4-year categories) and the full adjusted model included age (4-year categories), BMI (*kg/m*^2^), physical activity (MET *hr/day*), familial history of PCOS (yes/no) and total energy intake (*kcal/day*).

Models were mutually adjusted for a p-value <0.05 was used as the statistical evaluation tool.

## Results

The distribution of general characteristics of study participants among cases (n=281) and controls (n=472) has been shown previously (15). Compared to the controls, cases were significantly less physically active (48.6 MET *hr/d vs*. 59.8 MET *hr/day*; p<0.005) and had more energy intake (3215 *kcal/d vs*. 2489 *kcal/d*; p=0.027), higher BMI (31.2 *Kg/m*^2^ *vs*. 25.9 *Kg/m*^2^ ; p<0.001) and higher WC (93.3 *cm vs*. 80.1 *cm*; p<0.001). Cases had higher family history of PCOS (p<0.001). Sociodemographic data of participants across tertiles of healthy nutrient pattern is shown in table 1. There were no significant difference in distribution of these variables across tertiles of healthy dietary pattern.

**Table 1.** General characteristics of study participants according to the tertiles of healthy nutrient patterns

**Nutrient patterns**	**Tertiles of nutrient pattern**			p-value ^*^

	**1st**	**2nd**	**3rd**	

	**282**	**267**	**204**	
**No. Cases/No. Controls**	125/157	109/158	47/157	
**Age *(yr)* mean (SD)**	28.8 (7.3)	29.1 (7.5)	29.6 (7.2)	0.611
**Age at Menarche *(yr)* mean (SD)**	12.0 (2.1)	12.1 (2.1)	11.8 (1.8)	0.215
**Familial history of PCOS, no (%)**	64 (22.7)	58 (21.7)	46 (22.5)	0.105
**BMI (*kg/m*^2^) mean (SD)**	28.8 (5.1)	26.9 (4.3)	28.0(5.9)	0.082
**Waist circumference *(cm)* mean (SD)**	89.2(7.2)	79.6(6.9)	88.1(7.4)	0.035
**Physical activity (MET*/h/d)* mean (SD)**	49.3(5.5)	57.7(5.7)	53.9(6.1)	0.065
**Energy intake *(kcal/day)* mean (SD)**	3210(635)	2710(481)	2200(521)	0.025
**Cigarette smoker, no (%)**				0.211
**Never smoker**	120 (42.5)	137 (51.3)	109 (53.4)	
**Ex-smoker, pack-year <10**	25 (8.8)	24 (9.0)	17 (8.3)	
**Ex-smoker, pack-year ≥10**	28 (9.9)	27 (10.1)	20 (9.8)	
**Current smoker, pack-year <20**	69 (24.5)	59 (22.1)	49 (24.0)	
**Current smoker, pack-year ≥20**	40 (14.3)	20 (7.5)	9 (4.5)	
**Education, no (%)**				0.305
**Less than high school**	35 (12.4)	40 (14.9)	30 (14.7)	
**High school diploma**	169 (59.9)	131 (49.1)	115 (56.3)	
**Bachelor’s or higher**	78 (27.7)	96 (36.0)	59 (29.0)	
**Monthly family income, US $, no (%)**				0.852
**<300**	198 (70.2)	192 (72)	150 (73.5)	
**≥300**	84 (29.8)	75 (28.0)	54 (26.5)	
**Vitamin-mineral use, no (%)**	57 (20.2)	55 (20.6)	47 (23.0)	0.365

* ANOVA, SD: Standard deviation; BMI: Body mass index; LH: Luteinizing hormone; FSH: Follicle-stimulating hormone; PRL: Prolactin

Factor-loading matrix for the two retained factors is shown in table 2. These factors explained 58.3% of the total variance in the original dataset. Factor 1 explained 29.6% of the total variance and had high loadings for riboflavin (vitamin B _2 _), niacin (Vitamin B _3 _), pyridoxine (vitamin B _6 _), thiamin (vitamin B _1 _), magnesium, pantothenic acid (vitamin B _5 _), cobalamin (vitamin B _12 _), vitamin C, folate (vitamin B _9 _), vitamin D, total fiber, selenium, phosphorus, vitamin E, manganese, vitamin K, monounsaturated fatty acids, polyunsaturated fatty acids, potassium, and vegetable protein. Factor 2 displayed high loadings for sodium, cholesterol, saturated fatty acid, fat, biotin, carbohydrate, iron, fluoride, zinc, copper, calcium and animal protein.

**Table 2.** Factor-loading matrix for the nutrients representing the two major nutrient patterns from food frequency questionnaire data of participants ^*^

**Nutrients**	**Nutrient patterns**	

	**Factor 1**	**Factor 2**
**Vitamin B2 (Riboflavin)**	0.955	-
**Vitamin B3 (Niacin)**	0.946	0.317
**Vitamin B6 (Pyridoxine)**	0.933	-
**Vitamin B1 (Thiamin)**	0.825	0.361
**Magnesium**	0.821	0.347
**vitamin B5 (Pantothenic acid)**	0.781	0.329
**Vitamin B12 (Cobalamin)**	0.752	0.365
**Vitamin C**	0.730	0.325
**Vitamin B9 (Folate)**	0.723	-
**Vitamin D**	0.711	0.398
**Total fiber**	0.658	0.394
**Selenium**	0.621	-
**Phosphorus**	0.545	0.311
**Vitamin E**	0.523	-
**Manganese**	0.511	-
**Vitamin K**	0.501	-
**Monounsaturated fatty acids**	0.498	-
**Polyunsaturated fatty acids**	0.477	-
**Potassium**	0.459	-
**Vegetable protein**	0.411	-
**Sodium**	-	0.952
**Cholesterol**	-	0.868
**Saturated fatty acids**	0.487	0.855
**Fat**	0.406	0.771
**Biotin**	-	0.452
**Carbohydrate**	0.302	0.410
**Iron**	-	0.394
**Fluoride**	-	0.381
**Zinc**	-	0.356
**Copper**	-	0.325
**Calcium**	-	0.315
**Animal protein**	-	0.304
**Variance explained (%)**	29.6	28.7

* Estimates from principal component factor analysis performed on 32 nutrients. Absolute values of <0.3 are not shown in the table for simplicity

Table 3 shows the crude and multivariate adjusted means of the PCOS by tertiles of scores for each nutrient pattern. After adjusting for potential confounding factors, the highest tertile of the first pattern was associated with a lower risk of PCOS (OR: 0.48, 95% CI: 0.21–0.82, P for trend=0.002). Conversely, in the fully adjusted model, those in the highest tertile of the second pattern had a 2.38 times higher odds of PCOS (95% CI: 1.69–3.21; P for trend=0.012).

**Table 3.** Adjusted odds ratio (OR) estimates and 95% confidence intervals (CIs) for polycystic ovarian syndrome by nutrient patterns ^*^

**Nutrient patterns**	**Tertiles of nutrient patterns**			**p-value for trend**

	**1st**	**2nd**	**3rd**	
**Factor 1**				
No. Cases/No. Controls	125/157	109/158	47/157	
Minimal Model ^**^	1.00 (reference)	0.73 (0.52–1.02)	0.43 (0.37–0.77)	<0.001
Full Model ^***^	1.00 (reference)	0.76 (0.48–1.03)	0.48 (0.21–0.82)	0.002
**Factor 2**				
No. Cases/No. Controls	42/157	101/158	138/157	
Minimal Model ^**^	1.00 (reference)	1.56 (1.33–1.89)	2.87 (1.71–3.08)	0.009
Full Model ^***^	1.00 (reference)	1.49 (1.18–1.95)	2.38 (1.69–3.21)	0.012

* An unconditional logistic regression model,

** Adjusted for age (4-year categories),

*** Adjusted for age (4-year categories), BMI (*kg/m*^2^), total energy intake (*kcal/day*), familial history of PCOS and physical activity (MET *hr/day*)

## Discussion

To our knowledge, the present study, based on a valid and detailed FFQ, is the first case-control study to examine the relationship between nutrient pattern and PCOS risk. Our findings suggest that the first pattern (abundant in riboflavin, niacin, pyridoxine, thiamin, magnesium, pantothenic acid, cobalamin, vitamin C, folate, vitamin D, total fiber, selenium, phosphorus, vitamin E, manganese, vitamin K, monounsaturated fatty acids, poly-unsaturated fatty acids, potassium and vegetable protein) had a significant negative relationship with PCOS risk among a sample of Iranian women. In contrast, the second pattern (abundant in fat, animal protein, carbohydrate, cholesterol, saturated fatty acid, sodium, biotin, iron, copper, fluoride, zinc, and calcium) was positively associated with the risk of PCOS.

There are few studies evaluating the relationship between dietary factors and PCOS risk, and all of them assessed the isolated dietary components (22–24). Douglas et al. (8) have found that women with PCOS consume a greater amount of specific foods with a high glycemic index in comparison to healthy controls. Ahmadi et al. reported that women with PCOS had a diet with higher total energy and fat, saturated fat and poly-unsaturated fat (25). Omega-3 fatty acids supplementation (13) among women with PCOS had beneficial effects on their androgen profiles. A recent study suggests an inverse association between vitamin D status and metabolic disturbances in PCOS (26). The combination of low-glycemic-load foods and high-protein diet caused a significant increase in insulin sensitivity of PCOS patients (14). Improvement of androgen profiles was reported after intervention of vitamin D and calcium in overweight women with PCOS (27), however the results of other studies evaluating this effect are inconclusive (26). Altieri et al (28) did not find major differences between PCOS and normoandrogenic control women in regards of energy and macronutrient except for a higher intake of fibers and a lower percentage of energy from lipids by PCOS women. PUFAs modulated hormonal and lipid profiles and supplementation with long-chain n-3 PUFAs improves androgenic profiles in PCOS (29).

Most previous studies in PCOS patients have focused on the influence of an individual nutrient, whereas when an association with overall intake of a nutrient is observed, researchers may identify significant cumulative effects that may be too small to detect with isolated nutrients (30). The nutrient pattern approach is complementary to analyses using individual nutrients, which are limited by biologic interactions and colinearity among nutrients. The logic behind the nutrient pattern approach is that nutrients are not eaten separately but are eaten in the form of specified dietary patterns. In this study, the components of the first pattern could be found in a diet high in vegetarian foods, while the second pattern’s components are usually found in an animal based diet, so it seems that the interaction of nutrients in a diet high in vegetables can be protective for PCOS, while this interaction in an animal based diet prones women to PCOS.

The present study had several strengths; the most important of all is being the first study to assess the association of nutrient patterns with PCOS in a developing country. Studies in developing countries can provide unique opportunities to test the association between diet and disease (31). In addition where there is severe restriction in economic resources, food intake is strongly linked to income so that even small economic differences are directly reflected in diet which would increase the between-person variations. Furthermore, the different socio-economic backgrounds of people in developing countries compared to majority of people in the western world (mainly the status of women, family size, work participation, autonomy, parity, and access to the health care systems) influence the outcome as a confounder.

Moreover, the participation rate in this study was high, which was another positive point. Subjects whose disease was diagnosed three months before the interview at the most were registered in the present study as incident cases in order to reduce the possibility of recall bias. This is due to the fact that dietary data collected at the time of disease ascertainment might not truly reflect past intakes or intakes during the disease development and cases might have changed their diets due to the disease symptoms (protopathic bias). Controls were also carefully selected from patients only with conditions not related to diet or other major risk factors of PCOS. Using the factor analysis is another strength point of this study because the nutrient patterns derived from factor analysis capture the effects of the combination of many interacting and synergic nutrients and represent a significant biological vision to focus on; this might result in stronger or more consistent results which could be applied to various populations more easily so that factor analysis has emerged as a method for comprehensive estimation of disease risk as compared to traditional analyses of isolated nutrients (21).

Before the implications of our findings are considered, it is necessary to consider potential limitations and biases. First, several arbitrary but important decisions are involved in factor analysis approach, including the labeling of the components, the number of factors to extract, the method of rotation and even their interpretation (32). Second, measurement errors were inevitable because dietary intake was assessed using FFQ which might have led to underestimation of associations, however the FFQ used in this study has good validity and reproducibility among Iranian population (20) and we also excluded subjects who were under-/over-reporting their energy intakes. Third, recruitment of patients with other diseases as controls in the present study is a weakness because their exposure may not be representative of that in members of the study population who are at risk of becoming cases (33). In addition, control subjects might also have nutritional problems, which dilute the association of nutrient patterns and risk of PCO due to sharing the exposure; however we preferred hospital controls (as opposed to community controls) due to their higher participation and cooperation rates and to avoid selection bias. Furthermore, a range of control diagnoses was included to compensate for that limitation (33). Fourth, we enrolled participants aged 20–35 years old, which is late for diagnosis of PCOS. All of the cases were referred to our clinic with chief complaint of infertility. So, they might ignore their other symptoms previously, which indicates that they were careless about their health and were not generally healthy people with healthy lifestyle. Fifth, although residual confounding cannot be entirely ruled out due to imprecise measurements of important covariates, it is unlikely that errors in measuring the covariates were so extreme because the crude and multivariable results were essentially the same. Finally, it would have been ideal to match cases and controls for BMI values to be able to confidently compare them from a metabolic stand point. In this study we were concerned about overmatching problem which may cause to loss of efficiency, because the matching effect may narrow the exposure range.

## Conclusion

In conclusion the first nutrient pattern showed an inverse association and the second nutrient pattern showed a direct association with PCOS among Iranian women. However, case-control studies may prove an association but they do not demonstrate causation. As a result, these findings need to be confirmed in future prospective studies for etiological purposes to draw more conclusive results.
